# Hepatic iron overload and fibrosis in patients with beta thalassemia major after hematopoietic stem cell transplantation: A pilot study

**Published:** 2015-04-01

**Authors:** Ardeshir Ghavamzadeh, Mehrzad Mirzania, Naser Kamalian, Nahid Sedighi, Parisima Azimi

**Affiliations:** 1 Professor of Medicine, Hematology, Oncology and Stem Cell Transplantation Research Center, Tehran University of Medical Sciences, Tehran, Iran; 2 Hematologist and Medical Oncologist, Cancer Research Center, Cancer Institute, Tehran University of medical Sciences, Tehran, Iran; 3 Professor of Medicine, Tehran University of Medical Sciences, Tehran, Iran; 4 Associate Professor of Medicine, Advanced Diagnostic and Interventional Radiology, MSK research group, Tehran University of Medical Sciences, Tehran, Iran; 5 Tehran University of Medical Sciences, Tehran, Iran

**Keywords:** Hematopoietic Stem Cell Transplantation, Hepatic Iron Overload, Perls’ Prussian Blue Staining, Liver Fibrosis

## Abstract

Currently, hematopoietic stem cell transplantation (HSCT) is the only curative option for patients with beta-thalassemia major, but liver iron overload in these patients will not decrease and hepatic fibrosis may still progress despite successful HSCT.

Liver biopsy samples were taken from 14 patients (Out of 25 patients) who underwent HSCT. All patients met three criteria: negative HCV antibody, liver fibrosis in samples before HSCT and lack of regular treatment for iron overload after HSCT (Because patients did not consent to phlebotomy or they had not regular follow-up). We evaluated liver fibrosis and liver iron overload by a semi quantitative method, Perls’ Prussian blue staining, before and after HSCT.

HSCT was successful in all the patients. Liver iron overload did not change after transplant (P=0.61), but hepatic fibrosis progressed after transplant (P=0.01).

In patients with beta thalassemia major who previously had some degree of liver fibrosis, HSCT alone cannot reduce liver iron overload and liver fibrosis will increase. We recommend that regardless of the amount of iron overload in patients with beta thalassemia major that have shown some degree of fibrosis in their liver biopsy before transplantation, appropriate steps should be taken to reduce iron overload as soon as possible after successful transplantation.

## Introduction

 Beta-thalassemia is very common in Iran and is considered to be an endemic disease. Beta Thalassemia can be clinically divided into three forms: 1) a minor or trait form, 2) a major form that is dependent on blood transfusion, and 3) an intermediate form in which the patient has anemia, but usually becomes dependent on blood transfusion in the second decade of life. The main treatment for thalassemia major is regular blood transfusion with the major side effect of iron overload leading to severe complications within a few years and eventually to death if not treated. The mechanism of secondary cell damage due to intracellular iron overload is not clear, but it is believed to produce hydroxyl free radicals that lead to damage to cell lysosomes and mitochondria.^1^^,^^2^^,^^3^^,^^4^ The liver is the initial site of iron deposition, which results in fibrosis and then cirrhosis.^5^^,^^6^ In thalassemias that are dependent on blood transfusion, liver fibrosis directly depends on the patient’s age, number of blood units transfused, and liver iron concentration.^7^ Currently, hematopoietic stem cell transplantation is the only curative option for patients with  beta-thalassemia major.^11^ However, successful transplantation will not solve all patients’ problems, especially in the case of liver complications due to iron overload. Based on three criteria, Lucarelli et al. classified patients with thalassemia in terms of HSCT risks: the presence of hepatomegaly, the presence of liver fibrosis in the pretransplantation liver biopsy and a history of inadequate chelation therapy before HSCT. These three factors stratify patients into three groups: class 1, with none of the risk factors; class 2, having one or two of the risk factors; and class 3, with all three risk factors present. Event-free survival rates after transplantation are 90%, 81%, and 54% in classes 1, 2, and 3, respectively.^8^

 Liver biopsy and the semiquantitative measurement of liver iron concentration through histochemical staining methods such as Perls’ Prussian Blue Staining are the methods used for the assessment of the liver iron status.^6^^,^^9^^,^^10^ Another method is Hepatic Iron Concentration (HIC) expressed as milligrams of iron per gram of dry liver weight; it can also be expressed as µmole of iron per gram of dry liver weight, with normal values under 25 µmole/gr or 1.6 mg/gr. Values over 71 µmole/gr indicate liver iron overload.^3^^,^^6^^,^^12^^,^^13^^,^^14^

## SUBJECTS AND METHODS

 The patients with thalassemia major who had degrees of liver fibrosis in their liver biopsies before transplantation (Class 2 or 3) and had not received iron reduction treatments after successful transplantation were selected and invited for a second biopsy; all of them were negative for the HCV antibody test. The objectives of this study included determination of the degree of liver fibrosis and liver iron storage before and after HSCT. 

Out of 25 patients meeting the above-mentioned criteria, 4 patients with active evidence of chronic graft-versus-host disease (GVHD) and 7 patients who were not satisfied were excluded. The 14 remaining patients gave valid consent for liver biopsy. In 9 and 5 patients, stem cells were isolated from the peripheral blood and bone marrow donors, respectively. Informed consent was obtained from all patients and/or parents. Patients were admitted to the ward and liver biopsy was performed under ultrasound guidance. Information about patients is shown in Table 1.

 Iron is not demonstrable in the normal liver using standard histochemical  methods and the Perl’s histochemical method is widely used for this purpose because of its high sensitivity and specificity. This method also has the advantage of being relatively simple to perform. Hepatocytes with no increase in iron storage produce a pale blue color, but those with iron overload appear to have dark blue pigments in their cytoplasm, nuclear membrane, nucleolus, or sinusoidal spaces. Intensity of iron deposition in the liver is expressed as the grade or amount of the stained iron, and depends on the presence of iron deposits in acinar zones and portal structures, and also on the presence of fibrosis in the liver. The most common grading method of tissue iron overload is from 0 to 4+, but there are other methods in which the grade ranges from 0 to 5+.^9^^,^^10^

 Post-transplant liver biopsy slides were stained. The patients’ pre-transplant liver biopsy slides were then retrieved from the pathology archives. All slides were coded with numbers before being submitted to the

pathologist for review of iron and fibrosis status (Table 2).

## Results

 The mean time interval between bone marrow transplantation and liver biopsy after transplantation was 47.2 months (range: 9 to 102 months). In this study, 14 patients (5 males and 9 females) with a mean age of 11±6.2 years were investigated (age range: 4 to 25 years old). Of them, 6 were under and 8 were over 10 years of age. The source of stem cells was bone marrow in 5 and peripheral blood in 9 patients.

 The increasing fibrotic changes were significant (P=0.01), but iron variations did not show a statistically significant difference (P=0.61).

**Table 1:** Data of patients

**NO**	**Age at HSCT (year)**	**Sex**	**Risk Class**		**Conditioning** **regimen**		**Ferritin (ng/ml) before HSCT**		**Ferritin (ng/ml) After HSCT**	**Liver Iron Before HSCT (grade 0-5)**		**Liver Iron After HSCT (grade 0-5)**		**Liver Fibrosis Before HSCT (Score 0-5)**		**Liver Fibrosis After HSCT (Score 0-5)**		**Time from HSCT to Second Liver biopsy (mo)**			**Type of HSCT**
1	4	F		3		Bu/cy		350	170		0-1		1-2		1		1-2		102	BMT	
2	10	M		3		Bu/Cy		1910	1980		1-2		1-2		1-2		3		78	BMT	
3	8	M		2		Bu/Cy	2530		2150		3		3		2-3		3-4		73	BMT	
4	12	F		3		Bu/Cy		2200	2150		1-2		1-2		2		2-3		99	PBSCT	
5	25	M		3		Flu/ATG/Bu		900	950		4		2		2-3		3		20	PBSCT	
6	18	M		3		Flu/ATG/Bu		2730	2870		3-4		3-4		4		2-3		12	PBSCT	
7	18	M		2		Bu/Cy		1880	1710		3		4		2		2-3		30	PBSCT	
8	11	M		3		Bu/Cy		1375	1430		2-3		2-3		2		2-3		41	BMT	
9	15	F		3		Flu/ATG/Bu		2350	2260		4		4		4		4-5		14	PBSCT	
10	12	M		3		Flu/ATG/Bu		3150	2920		4		3-4		1		3		52	PBSCT	
11	6	F		3		Bu/Cy		1500	1670		1-2		2		1-2		2		9	BMT	
12	4	M		2		Bu/Cy		1350	1370		2-3		2		1-2		3		64	PBSCT	
13	5	F		2		Bu/Cy		840	610		2		3		1		1		38	PBSCT	
14	6	M		2		Bu/Cy		2100	1900		2-3		2-3		1-2		2-3		30	PBSCT	

**HSCT**: Hematopoietic Stem Cell Transplantation, **F**: Female, **M**: Male, **Bu/Cy**: Busulfan/Cyclophosphamide, **BMT**: Bone Marrow Transplantation, **PBSCT**: Peripheral Blood Stem Cell Transplantation, **Flu/ATG/Bu**: Fludarabine/Anti-thymocyte globulin/Busulfan

The comparison was performed using the Wilcoxon signed ranks test.

 Changes in fibrosis were directly correlated with the time of biopsy, but the correlation was not statistically significant. The later the biopsy was performed, the more fibrosis was reported but the correlation was not significant due to the small sample size of the study (Pearson correlation coefficient =0.44, P=0.11). However, the Spearman correlation test showed no linear correlation between iron changes and the time of biopsy (Spearman correlation coefficient=0.005, P=0.99). 

The Mann-Whitney test showed no significant difference in iron changes between males and females (P=0.15 and 0.08, respectively). 

 Additionally, according to the Mann-Whitney test, there was no significant difference between patients <10 and those ≥10 years of age in terms of iron changes (P=0.28 and 0.78, respectively). The Mann-Whitney test showed no significant difference between transplantation using bone marrow and transplantation using the peripheral blood in terms of iron and fibrosis changes (P=0.36 and 0.7, respectively). 

**Table 2:** Liver iron and fibrosis score before and after Hematopoietic Stem Cell Transplantation

		**Age groups**		**Gender**		**Stem cell Source**	
		**<10y**	**>=10y**	**Female**	**Male**	**BMT**	**PBSCT**
**Iron**							
	**Deteriorated**	3 (50%)	1 (12.5%)	3 (60%)	1 (11.1%)	2 (40%)	2 (22.2%)
	**No change**	2 (33.3%)	5 (62.5%)	2 (40%)	5 (55.6%)	3 (60%)	4 (44.4%)
	**Improved**	1 (16.7%)	2 (25%)	0 (0%)	3 (33.3%)	0 (0%)	3 (33.3%)
**Fibrosis**							
	**Deteriorated**	5 (83.3%)	7 (87.5%)	4 (80%)	8 (88.9%)	5 (100%)	7 (77.8%)
	**No change**	1 (16.7%)	0 (0%)	1 (20%)	0 (0%)	0 (0%)	1 (11.1%)
	**Improved**	0 (0%)	1 (12.5%)	0 (0%)	1 (11.1%)	0 (0%)	1 (11.1%)

**HSCT:** Hematopoietic Stem Cell Transplantation, **F**: Female, **M**: Male, **Bu/Cy**: Busulfan/Cyclophosphamide, **BMT**: Bone Marrow Transplantation, **PBSCT**: Peripheral Blood Stem Cell Transplantation, **Flu/ATG/Bu**: Fludarabine/Anti-thymocyte globulin/Busulfan

## Discussion

 Iron depletion by phlebotomy after HSCT can cause cirrhosis regression in patients with iron overload.^5^ The use of iron chelators such as Deferoxamine after HSCT has the same effect.^15^ Deferoxamine should not be administered immediately after transplantation because it can interfere with engraftment.

 In one study in Pesaro, 48 patients who had been regularly followed-up after transplantation for homozygous beta thalassemia were enrolled in a program of regular phlebotomy. Patients were eligible for this study if they were in prognostic classes 2 or 3 before transplantation; the 2-year follow-up examinations showed serum ferritin levels greater than 2,000 μg/L and the 2-year post-transplant liver biopsies showed moderate or severe iron overload without phlebotomy. In this study thirty-five (85%) patients were hepatitis C seropositive. The results of the study showed that serum ferritin and HIC were reduced after repeated phlebotomy (P Value= 0.0001), but the degree of hepatic fibrosis was not significantly changed (P Value 0.18).^8^ In this study, fibrosis and iron overload before HSCT were not compared with the values after HSCT (prior to phlebotomy).

In our center, most patients with beta thalassemia major were treated with phlebotomy after successful transplantation, but some of them had not received this treatment, because did not consent to phlebotomy or they had not regular follow-up.

 The main selection criterion in our study was liver fibrosis before transplantation (prognostic classes 2 or 3) regardless of the severity of iron accumulation in the liver or serum ferritin level. In this study we have shown that liver fibrosis alone before HSCT (with any degree of hepatic iron overload) can cause liver fibrosis progression after HSCT.

In patients with mild to moderate iron overload, a gradual spontaneous decrease of iron overload after HSCT was almost sufficient to normalize iron levels^16^, but this did not occur in patients with severe iron overload at the time of transplant; in these patients, there may be progression of liver damage after HSCT.^17^^,^^18^ Our investigation has shown, however, that liver fibrosis before HSCT in patients with β-thalassemia is a more important factor.

 A lack of regression of hepatic fibrosis after phlebotomy^8^ and the progression of liver fibrosis after transplantation shown in our study, highlight the importance of preventive phlebotomy.

Combining the four criteria in all patients (lack of iron overload treatment after successful HSCT, the presence of fibrosis in biopsy samples before transplantation, HCV antibody negativity and no active evidence of GVHD) limited the number of samples available for this study.

 Another point that should be considered in the study was the assessment of iron overload using Perl’s histochemical tissue staining. We used this method because quantitative measurement (HIC) was not available in our country. However, the results of our study are supported by studies that have used HIC. In other words, non-depletion of the excess iron by phlebotomy or iron chelators after HSCT will result in stabilization of the amount of liver iron.^6^^,^^7^^,^^8^^,^^9^^, ^^12^

 According to our results, patients who had received transplantation at least 9 months earlier had liver fibrosis progression. 

## CONCLUSION

 We recommend that if HSCT is successful in a patient with any degree of liver fibrosis before transplantation (irrespective of the severity of iron overload), the total body iron load should be reduced with phlebotomy as soon as possible.
